# Metaheuristic Based Scheduling Meta-Tasks in Distributed Heterogeneous Computing Systems

**DOI:** 10.3390/s90705339

**Published:** 2009-07-07

**Authors:** Hesam Izakian, Ajith Abraham, Václav Snášel

**Affiliations:** 1 Islamic Azad University, Ramsar Branch, Ramsar, Iran; E-Mail: hesam.izakian@gmail.com; 2 Machine Intelligence Research Labs (MIR Labs), Auburn, Washington 98071-2259, USA; http://www.mirlabs.org; 3 Norwegian Center of Excellence, Center of Excellence for Quantifiable Quality of Service, Norwegian University of Science and Technology, O.S. Bragstads plass 2E, N-7491 Trondheim, Norway; 4 Faculty of Electrical Engineering and Computer Science VSB-Technical University of Ostrava, Czech Republic; E-Mail: vaclav.snasel@vsb.cz

**Keywords:** distributed heterogeneous computing systems, particle swarm optimization, scheduling

## Abstract

Scheduling is a key problem in distributed heterogeneous computing systems in order to benefit from the large computing capacity of such systems and is an NP-complete problem. In this paper, we present a metaheuristic technique, namely the Particle Swarm Optimization (PSO) algorithm, for this problem. PSO is a population-based search algorithm based on the simulation of the social behavior of bird flocking and fish schooling. Particles fly in problem search space to find optimal or near-optimal solutions. The scheduler aims at minimizing makespan, which is the time when finishes the latest task. Experimental studies show that the proposed method is more efficient and surpasses those of reported PSO and GA approaches for this problem.

## 1. Introduction

A distributed heterogeneous computing (HC) system consists of a distributed suite of different high-performance machines, interconnected by high-speed networks, to perform different computationally intensive applications that have various computational requirements. Heterogeneous computing systems range from diverse elements or paradigms within a single computer, to a cluster of different types of PCs, to coordinated, geographically distributed machines with different architectures (e.g., Grids [1]).

To exploit the different capabilities of a suite of heterogeneous resources effectively and satisfy users with high expectations for their applications, a crucial problem that needs to be solved in the framework of HC is the scheduling problem.

Optimal scheduling involves mapping a set of tasks to a set of resources to efficiently exploit the capabilities of such systems. As mentioned in [2], optimal mapping tasks to machines in an HC suite is an NP-complete problem and therefore the use of heuristics is one of the suitable approaches. According to the type of tasks being scheduled, the scheduling problem can be classified into two types: scheduling meta-tasks and scheduling a directed acyclic graph (DAG) composed of communicating tasks. In this paper, we consider meta-task scheduling problem which involve allocation of a set of independent tasks from different users to a set of computing resources.

In recent years some works have been done using pure heuristics to find near-optimal solutions. These heuristics are fast, straightforward and easy to implement. Some popular and efficient pure heuristics are Sufferage [3], min-min [4], max-min [4], LJFR-SJFR [5], min-max [6], etc. Also, to improve the quality of solutions, meta-heuristics have been presented for task scheduling problem. The most popular of meta-heuristic algorithms are genetic algorithm (GA) [7], simulated annealing (SA) [8], ant colony optimization (ACO) [9] and particle swarm optimization (PSO) [10].

Ritchie and Levine [11] used a hybrid ant colony optimization for scheduling in HC systems. In this method, authors combined ant colony optimization with local and tabu search to find shorter schedules. Yarkhan and Dongarra [12] used simulated annealing approach for grid job scheduling. Page and Naughton [13] used a genetic algorithm method for scheduling HC systems. In this method the scheduling strategy operates in a dynamically changing computing resource environment and adapts to variable communication costs and variable availability of processing resources. Braun *et al*. [14] described eleven heuristics and compared them on different types of HC environments. The authors illustrated that the GA scheduler can obtain better results in comparison with others.

Xhafa *et al*. [15] used Genetic Algorithm-based schedulers for computational grids and most of GA operators are implemented and compared to find the best GA scheduler for this problem. In [16] the authors also focused on Struggle Genetic Algorithms and their tuning for scheduling of independent jobs in computational grids. Hash-based implementations of the struggle Genetic operator for the GAs were proposed. Abraham *et al*. [17] used a fuzzy particle swarm optimization and Izakian *et al*. [18] used a discrete version of particle swarm optimization for scheduling problem.

Xhafa *et al*. [19] exploited the capabilities of Cellular Memetic Algorithms (CMA) for obtaining efficient batch schedulers for grid systems. Authors implemented and studied several methods and operators of CMA for the job scheduling in grid systems. Abraham *et al*. [20] illustrated the usage of several nature inspired meta-heuristics (SA, GA, PSO, and ACO) for scheduling jobs in computational grids using single and multi-objective optimization approaches. Also Xhafa and Abraham [21] have reviewed the most important concepts from grid computing related to scheduling problems and their resolution using heuristic and meta-heuristic approaches. The authors identified different types of scheduling based on different criteria, such as static vs. dynamic environment, multi-objectivity, adaptivity, etc.

Different criteria can be used for evaluating the efficiency of scheduling algorithms, the most important of which is makespan. Makespan is the time when an HC system finishes the latest task. An optimal schedule will be the one that minimizes the makespan.

PSO is an algorithm that follows a collaborative population-based search model and has been applied successfully to a number of problems, including standard function optimization problems [22], solving permutation problems [23] and training multi-layer neural networks [24] and its use is rapidly increasing. A PSO algorithm contains a swarm of particles in which each particle includes a potential solution. In contrast to evolutionary computation paradigms such as Genetic Algorithm, a swarm is similar to a population, while a particle is similar to an individual. The particles fly through a multidimensional search space in which the position of each particle is adjusted according to its own experience and the experience of its neighbors. PSO system combines local search methods (through self experience) with global search methods (through neighboring experience), attempting to balance exploration and exploitation [25].

In this paper, we present a version of particle swarm optimization approach for scheduling meta-tasks in HC systems and the goal of scheduler is to minimize the makespan. In order to evaluate the performance of the proposed method, it is compared with genetic algorithm that presented in [14] for scheduling tasks in HC systems and continuous PSO that presented in [25] for task assignment problem. The experimental results show the presented method is more efficient and can be effectively used for HC systems scheduling. The remainder of this paper is organized in the following manner. In Section 2, we formulate the problem, in Section 3 the PSO paradigm is briefly discussed, Section 4 describes the proposed method and Section 5 reports the experimental results. Finally Section 6 concludes this work.

## 2. Problem Definition

An HC environment is composed of computing resources where these resources can be a single PC, a cluster of workstations or a supercomputer. Let *T* = {*T*_1_, *T*_2_,…,*T*_n_} denote the set of tasks that in a specific time interval is submitted to HC system. Assume the tasks are independent of each other (with no inter-task data dependencies) and preemption is not allowed (they cannot change the resource they have been assigned to). Also assume at the time of submitting these tasks, *m* machines *M* = {*M*_1_, *M*_2_,…,*M*_m_} are within the HC environment. In this paper it is assumed that each machine uses the First-Come, First-Served (FCFS) method for performing the received tasks. We assume that each machine in HC environment can estimate how much time is required to perform each task. In [14] Expected Time to Compute (ECT) matrix is used to estimate the required time for executing a task in a machine. An ETC matrix is an *n* × *m* matrix in which *n* is the number of tasks and *m* is the number of machines. One row of the ETC matrix contains the estimated execution time for a given task on each machine. Similarly one column of the ETC matrix consists of the estimated execution time of a given machine for each task. Thus, for an arbitrary task *T_j_* and an arbitrary machine *M_i_*, *ETC* (*T_j_*, *M_i_*) is the estimated execution time of *T_j_* on *M_i_*. In ETC model we take the usual assumption that we know the computing capacity of each resource, an estimation or prediction of the computational needs of each task, and the load of prior work of each resource.

Assume that *C_i,j_* (*i* ∈ {1,2,…*m*}, *j* ∈ {1,2,…*n*}) is the execution time for performing *j*th task in *i*th machine and *W_i_* (*i* ∈ {1,2,…*m*}is the previous workload of *M_i_*, then (1) shows the time required for *M_i_* to complete the tasks included in it. According to the aforementioned definition, makespan can be estimated using (2):

(1) $$\underset{\forall \;\mathrm{task}\;j\;\mathrm{allocated}\;\mathrm{to}\;\mathrm{machine}\;i}{\;\;\;\sum C_{\mathrm{ij}}+W_{i}}$$

(2) $$\mathrm{makespan}=\text{max}\{\underset{\forall \;\mathrm{task}\;j\;\mathrm{allocated}\;\mathrm{to}\;\mathrm{machine}\;i}{\;\;\;\sum C_{\mathrm{ij}}+W_{i}}\},\;i\in \{1,2,...,m\}$$

In this paper the goal of scheduler is to minimize makespan.

## 3. Particle Swarm Optimization

**Figure 1.**
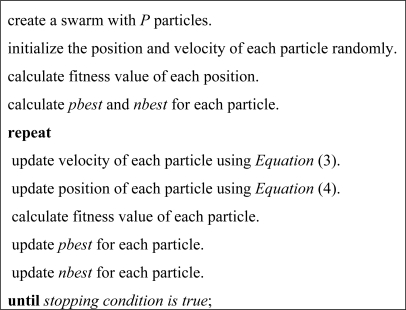
Pseudo-code of particle swarm optimization approach.

Particle swarm optimization (PSO) is a population based stochastic optimization technique inspired by bird flocking and fish schooling originally designed and introduced by Kennedy and Eberhart [10] in 1995. The algorithmic flow in PSO starts with a population of particles whose positions, which represent the potential solutions for the studied problem, and velocities are randomly initialized in the search space. In each iteration, the search for optimal position is performed by updating the particle velocities and positions. Also in each iteration, the fitness value of each particle’s position is determined using a fitness function. The velocity of each particle is updated using two best positions, personal best position and neighborhood best position. The personal best position, *pbest*, is the best position the particle has visited and *nbest* is the best position the particle and its neighbors have visited since the first time step. Based on the size of neighborhoods two PSO algorithms can be developed. When all of the population size of the swarm is considered as the neighbor of a particle *nbest* is called global best (*gbest*) and if the smaller neighborhoods are defined for each particle, then *nbest* is called local best (*lbest*). *gbest* uses the star neighborhood topology and *lbest* usually uses ring neighborhood topology. There are two main differences between *gbest* and *lbest* with respect to their convergence characteristics. Due to the larger particle interconnectivity of the *gbest* PSO it converges faster than the *lbest* PSO, but *lbest* PSO is less susceptible to being trapped in local optima. A particle’s velocity and position are updated as follows:

(3) $$V_{k}=V_{k}+c_{1}r_{1}({\mathrm{pbest}}_{k}-X_{k})+c_{2}r_{2}({\mathrm{nbest}}_{k}-X_{k});\;k=1,2,\ldots P$$

(4) $$X_{k}=X_{k}+V_{k}$$

where *c*_1_ and *c*_2_ are positive constants, called acceleration coefficients which control the influence of *pbest* and *nbest* on the search process, *P* is the number of particles in the swarm, *r*_1_ and *r*_2_ are random values in range [0, 1] sampled from a uniform distribution. Figure 1 shows the pseudo-code of particle swarm optimization approach.

## 4. PSO for Task Scheduling in HC Systems

In this section, we propose a version of particle swarm optimization for HC system scheduling. In this method, we add a heuristic to PSO. Particles need to be designed to present a sequence of tasks in available machines in HC system. Also the velocity has to be redefined.

### 4.1. Particles Encoding

One of the key issues in designing a successful PSO algorithm is the representation step, i.e. finding a suitable mapping between problem solution and PSO particle. In this paper each particle’s position is encoded in an *n*-dimensional search space in which *n* is the number of tasks to be scheduled. The value of each dimension is a natural number included in range [1, *m*] indicating the machine number, in which *m* is the number of available machines in HC system at the time of scheduling. Assume that *X_k_* = {*X_k_*_1_, *X*_k2_,…,*X*_kn_} shows the position of *k*th particle; *X_kj_* indicates the machine where task *T_j_* is assigned by the scheduler in this particle. Note that in this encoding method a machine number can appear more than once in a particle.

Since *pbest* and *nbest* are two positions that include the personal best position and neighborhood best position of each particle, therefore the *pbest* and *nbest* encoding is similar to the particle’s position. Also in this paper we used start topology for *nbest* (*gbest* PSO).

In our proposed method, velocity of each particle is considered as an *m* × *n* matrix whose elements are real numbers in range [1, *V*_max_]. Formally if *V_k_* is the velocity matrix of *k*th particle, then:

(5) $$V_{\mathrm{kij}}\in [1,\;V_{\text{max}}]\;(\forall i,\;j)\;,i\in \{1,2,...m\},\;j\in \{1,2,...,n\}$$

### 4.2. Updating Particles

**Figure 2.**
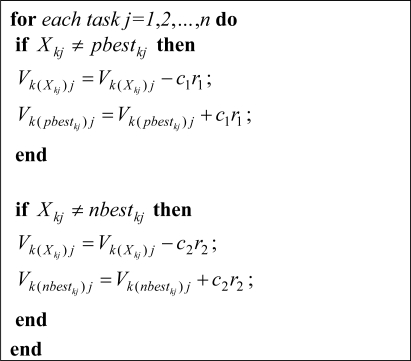
Velocity updating method.

In our proposed method similar to classic PSO, at first the particle’s velocity is updated and then it is used for updating the particles’ position. Figure 2 shows the pseudo-code for updating velocity matrix for particle *k*.

In this figure *c*_1_ and *c*_2_ are acceleration coefficients, *r*_1_ and *r*_2_ are random values in range [0, 1] sampled from a uniform distribution and *X_k_* is the position of particle *k*. For updating particle’s position we use the updated velocity matrix and a heuristic, *η* which adds an explicit bias towards the most attractive solutions and is a problem-dependent function. In our proposed method for updating a particle’s position, for each task, the probability of its performing on various machines is calculated according to (6):

(6) $$p_{\mathrm{kij}}=\frac{V_{\mathrm{kij}}\times {\left[\eta_{\mathrm{kij}}\right]}^{\beta }}{\sum_{l=1,2,...m}V_{\mathrm{klj}}\times {\left[\eta_{\mathrm{klj}}\right]}^{\beta }}$$

where *p_kij_* is the probability of performing task *T_j_* on machine *M_i_* in particle *k*, and *η_kij_* represents a priori effectiveness of performing task *T_j_* on machine *M_i_* in particle *k*. Since in this paper we aim at minimizing makespan, *η_kij_* is obtained using (7):

(7) $$\eta_{\mathrm{kij}}=\left(\frac{1}{{\mathrm{CT}}_{\mathrm{kij}}}\right)$$

in which *CT_kij_* is the completion time of task *T_j_* on machine *M_i_* in particle *k* and can be obtained according to the workload of machine *M_i_* plus required time for executing task *T_j_* on machine *M_i_*.

After obtaining the *p_kij_*, ∀ *i* = 1,2,…*m*, we can select a machine for task *T_j_* in particle *k* according to (8). In this equation *r*_0_ ∈ [0, 1] is a user specified parameter and *r* is a random number in range (0,1) sampled from the uniform distribution:

(8) $$M_{i}\leftarrow \{\begin{array}{ll} {\text{arg max}}_{l=1,2,..m}\;p_{\mathrm{klj}} & \text{if}\;r\leq r_{0}\\ \mathrm{roulette}\;\mathrm{wheel}\;\mathrm{selection}, & \text{otherwise} \end{array}$$

### 4.3. Fitness Evaluation

Since in this paper the makespan is used to evaluate the performance of scheduler, the Fitness value of each solution can be estimated using (9):

(9) $$\text{fitness}=\frac{1}{\mathrm{makespan}}$$

**Figure 3.**
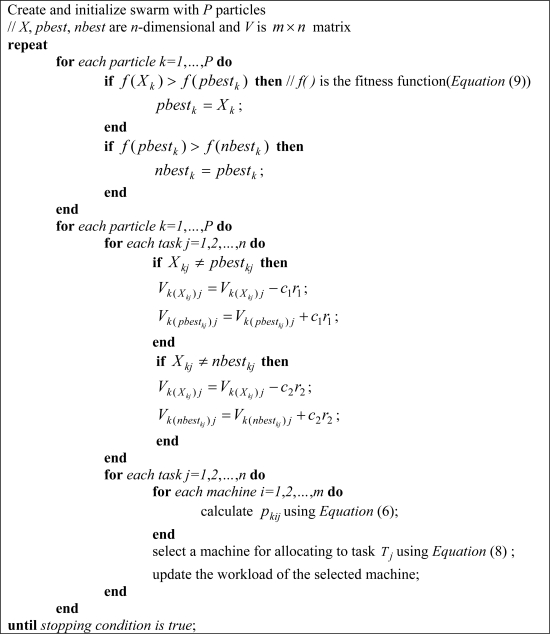
Pseudo-code of the proposed method.

Figure 3 shows the pseudo-code of our proposed method.

## 5. Experimental Results

In order to evaluate the performance of the proposed method, the approach was compared with a genetic algorithm [14] and continuous PSO [25] for task assignment problem in multiprocessor systems. The goal of scheduler in these methods is to minimize the makespan. These methods are implemented using VC++ and run on a Pentium IV 3.2 GHz PC. In order to optimize the performance of the proposed method and proposed PSO in [25] and GA in [14], fine tuning has been performed and best values for their parameters are selected. For the proposed method the following ranges of parameter values were tested: *c*_1_ and *c*_2_ = [1, 3], *P* = [10, 100], *V*_max_ = [10, 100], *β* = [0.1, 4] and *r*_0_ = [0.1, 0.9]. Based on experimental results the proposed PSO algorithm performs best under the following settings: *c*_1_ = *c*_2_ = 2.0, *P* = 50, *V*_max_ = 40, *β* = 1.0, *r*_0_ = 0.8. Also we used the benchmark that proposed in [14] for simulating the HC environment.

The simulation model in [14] is based on expected time to compute (ETC) matrix for 512 tasks and 16 machines. The instances of the benchmark are classified into 12 different types of ETC matrices according to the three following metrics: task heterogeneity, machine heterogeneity, and consistency. In ETC matrix, the amount of variance among the execution times of tasks for a given machine is defined as task heterogeneity. Machine heterogeneity represents the variation that is possible among the execution times for a given task across all the machines. Also an ETC matrix is said to be consistent whenever a machine *M_i_* executes any task *T_j_* faster than machine *M_k_*; in this case, machine *M_i_* executes all tasks faster than machine *M_k_*. In contrast, inconsistent matrices characterize the situation where machine *M_i_* may be faster than machine *M_k_* for some tasks and slower for others. Partially-consistent matrices are inconsistent matrices that include a consistent sub-matrix of a predefined size [14]. Instances consist of 512 tasks and 16 machines and are labeled as u-x-yy-zz as follows:

- u means uniform distribution used in generating the matrices.
- x shows the type of inconsistency; c means consistent, i means inconsistent, and p means partially-consistent.
- yy indicates the heterogeneity of the tasks; hi means high and lo means low.
- zz represents the heterogeneity of the machines; hi means high and lo means low.

In our experiment, the initial population for the compared methods is generated using two scenarios: (a) randomly generated particles from a uniform distribution, and (b) one particle using the min-min heuristic (that can achieve a very good reduction in makespan [6,14]) and the others are random solutions.

The statistical results of over 50 independent runs are compared in Table 1 for scenario (a). In the table the first column indicates the instance name, the second, third, and fourth columns indicate the makespan achieved by GA [14], PSO [25] and our proposed method respectively.

As shown in Table 1, the proposed PSO approach achieved best results in all instances. Also our method has a large amount of reduction in makespan in all instances; this is because of using heuristic *η* in the proposed method that minimizes makespan efficiently.

**Figure 4.**
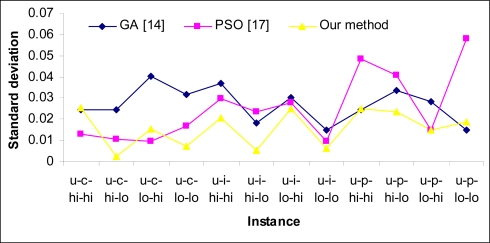
Standard deviation in scenario (a).

**Figure 5.**
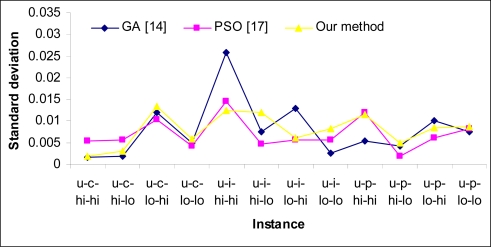
Standard deviation in scenario (b).

**Figure 6.**
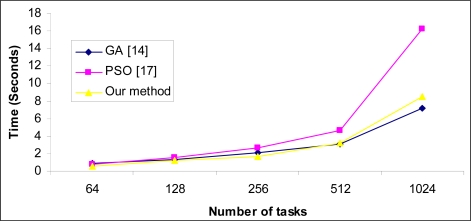
Comparison of convergence time between different methods.

Table 2 shows the statistical results of over 50 independent runs in scenario (b). As shown in this table, the min-min heuristic can obtain a good reduction in makespan. In this scenario our method surpasses others in most instances, except those with low heterogeneity in tasks and machines. Figures 4 and 5 show the standard deviation of the compared methods for scenario (a) and scenario (b), respectively. As shown in Figure 4, the proposed method has the lowest standard deviation; this is because of the use of heuristic *η* in our method. Figure 5 also shows that the magnitude of standard deviation is decreased in scenario (b) thanks to the use of the min -min heuristic. In this scenario, the PSO approach proposed in [25] has lowest standard deviation in most instances and our method has admissible standard deviation too. Figure 6 shows a comparison of CPU times required to achieve results between compared methods. It is evident that the proposed method needs the lowest time for convergence in most cases, but by increasing the number of tasks and problem search space, the time for achieving results is increased in the proposed method rather than GA and in case of 1,024 tasks, the GA scheduler needs lowest time for convergence.

## 6. Conclusions

To exploit the different capabilities of a suite of heterogeneous resources effectively and satisfy users with high expectations for their applications, a crucial problem that needs to be solved in the framework of HC is the scheduling problem. In this paper, we have combined particle swarm optimization approach with heuristic for scheduling tasks in distributed heterogeneous systems to minimize makespan. The performance of the proposed method was compared with GA and continuous PSO through carrying out exhaustive simulation tests and different settings. Experimental results show that our method surpasses other proposed techniques in most cases. In the future, we will formulate the proposed method for minimizing makespan and flowtime as a multi-objective problem.

## Figures and Tables

**Table 1.** Comparison of statistical results between GA [14], PSO[25] and the proposed method for scenario (a).

**Instance**	**GA[14]**	**PSO[25]**	**Proposed method**

u-c-hi-hi	21508486	13559696	**10173411**
u-c-hi-lo	236653	223008	**191878**
u-c-lo-hi	695320	463241	**371355**
u-c-lo-lo	8021	7684	**6379**
u-i-hi-hi	21032954	23114941	**6642987**
u-i-hi-lo	245107	286339	**149997**
u-i-lo-hi	693461	849702	**228971**
u-i-lo-lo	8281	9597	**4496**
u-p-hi-hi	21249982	22073358	**8325090**
u-p-hi-lo	242258	266825	**162601**
u-p-lo-hi	712203	772882	**293335**
u-p-lo-lo	8233	8647	**5213**

**Table 2.** Comparison of statistical results between the proposed method and others in scenario (b).

**Instance**	**Min-min**	**GA[14]**	**PSO[25]**	**Proposed method**

u-c-hi-hi	8145395	7892199	7867899	**7796844**
u-c-hi-lo	164490	161634	161437	**160639**
u-c-lo-hi	279651	276489	274636	**266747**
u-c-lo-lo	5468	**5292**	5322	5309
u-i-hi-hi	3573987	3496209	3560537	**3220459**
u-i-hi-lo	82936	81715	81915	**80754**
u-i-lo-hi	113944	112703	113171	**108597**
u-i-lo-lo	2734	**2636**	2680	2644
u-p-hi-hi	4701249	4571336	4580666	**4462357**
u-p-hi-lo	106322	104854	104987	**103794**
u-p-lo-hi	157307	153970	154933	**150375**
u-p-lo-lo	3599	**3449**	3473	3461
